# Identification of the Function of the Pathogenesis-Related Protein GmPR1L in the Resistance of Soybean to *Cercospora sojina* Hara

**DOI:** 10.3390/genes14040920

**Published:** 2023-04-15

**Authors:** Yeyao Du, Nooral Amin, Naveed Ahmad, Hanzhu Zhang, Ye Zhang, Yang Song, Sujie Fan, Piwu Wang

**Affiliations:** 1 College of Agronomy, Jilin Agricultural University, Changchun 130118, China; 2 Joint Center for Single Cell Biology, Shanghai Collaborative Innovation Center of Agri-Seeds, School of Agriculture and Biology, Shanghai Jiao Tong University, Shanghai 200240, China; 3 Jilin Provincial Seed Management Station, Changchun 130033, China; 4 Northeast Institute of Geography and Agroecology, Chinese Academy of Sciences, Changchun 130118, China

**Keywords:** soybean, *GmPR1L*, *Cercospora sojina* Hara, defense enzyme activity, relative expression level, agronomic traits

## Abstract

Pathogenesis-related proteins, often used as molecular markers of disease resistance in plants, can enable plants to obtain systemic resistance. In this study, a gene encoding a pathogenesis-related protein was identified via RNA-seq sequencing analysis performed at different stages of soybean seedling development. Because the gene sequence showed the highest similarity with PR1L sequence in soybean, the gene was named *GmPR1-9-like* (*GmPR1L*). *GmPR1L* was either overexpressed or silenced in soybean seedlings through *Agrobacterium*-mediated transformation to examine the resistance of soybean to infection caused by *Cercospora sojina* Hara. The results revealed that *GmPR1L*-overexpressing soybean plants had a smaller lesion area and improved resistance to *C. sojina* infection, whereas *GmPR1L*-silenced plants had low resistance to *C. sojina* infection. Fluorescent real-time PCR indicated that overexpression of *GmPR1L* induced the expression of genes such as *WRKY*, *PR9*, and *PR14*, which are more likely to be co-expressed during *C. sojina* infection. Furthermore, the activities of SOD, POD, CAT, and PAL were significantly increased in *GmPR1L*-overexpressing soybean plants after seven days of infection. The resistance of the *GmPR1L*-overexpressing lines OEA1 and OEA2 to *C. sojina* infection was significantly increased from a neutral level in wild-type plants to a moderate level. These findings predominantly reveal the positive role of *GmPR1L* in inducing resistance to *C. sojina* infection in soybean, which may facilitate the production of improved disease-resistant soybean cultivars in the future.

## 1. Introduction

Soybean (*Glycine max*) is an important economic and oil-bearing crop worldwide. Its production is affected by various factors, such as limited land resources and uncontrollable environmental changes, with the most serious factor being the frequent occurrence of diseases [1]. In recent years, soybean yield reduction caused by diseases is approximately 5–10%, and up to 30–50% [2]. Viral, bacterial, and fungal diseases are common in soybean. Fungal diseases have many subtypes, a high incidence, and severe harmful effects. Long-term use of pesticides is usually adopted to control these dangerous diseases. However, it not only increases the cost of soybean production but also causes serious pollution to the soil environment. Moreover, diseases are difficult to be controlled once pathogens develop resistance. The most direct and effective way of reducing the occurrence of diseases is to cultivate new resistant germplasm. Compared with traditional breeding methods, transgenic technology can overcome the problem of distant hybridization incompatibility and offers abundant target gene resources, greatly shortening the breeding period. Therefore, cultivating new disease-resistant varieties through genetic engineering is an effective strategy. 

Pathogenesis-related proteins (PRPs) are produced by plants under adversity and stress. These proteins play an important role in defense mechanisms and can be induced by pathogenic agents, chemical reagents, and plant hormones [3]. PRPs are widely found in many plants. They can participate in disease resistance through cell wall thickening, antibacterial activity, signal transduction, and many other mechanisms [2]. The primary functions of PRPs include degradation of toxins, elimination of pathogens, and binding to or inhibition of viral coat proteins [4]. PRPs are mainly divided into 19 subclasses according to their sources, differences in electrophoretic mobility, amino acid sequence homology, and association with serum [5]. Of the different gene classes, *PR1* family genes are most abundant, conservative, and stable. They are an indispensable part of the PRP family, and most proteases have no effect on them [6]. PR1 proteins participates in defense mechanisms in plants and have strong antifungal activity. Several studies have reported that PR-1 proteins interact with other proteins in plants. Ghorbel et al. demonstrated that durum wheat *PR1* (*TdPR1.2*) interacted with calmodulin in a calcium-dependent manner and this interaction enhanced the catalytic activity of *TdPR1.2* in vitro, especially in the presence of Mn^2+^ cations. OsSAP1, a stress-associated protein in rice, interacts with aminotransferases and *PR1a* called OsScP to regulate abiotic stress response in rice [7].

As early as 1980, Antoniw et al. were the first to identify PR1 proteins from tobacco extracts. PR1 proteins are involved in the response to various biotic and abiotic stresses and are promising marker genes for systemic acquired resistance (SAR). PR1 proteins are classified as acidic and basic. Acidic proteins are secreted in the intercellular space, whereas basic proteins are secreted in the cellular fluid. With the increasing number of studies on the PR1 family, PR1 proteins have been identified from rice [8], corn [9], and grape [10]. Previous studies have demonstrated that PR1 proteins are easily induced by pathogenic agents and salicylic acid and can accumulate up to tens of thousands of times in infected plant tissues, accounting for 1.2–2.5% of the total protein content in the leaves of the whole plant [9]. Exogenous SA can induce the development of SAR and expression of *PR1* genes in model plants [10]. Some studies have demonstrated that *PR1* genes in some plants can induce a certain level of resistance to pathogens. Alexander et al. [11] transferred the *PR1-a* gene into tobacco and found that it enhanced the resistance to *Phytophthora* and *Peronospora* infections in a highly constitutive expression-dependent manner. Niki et al. [12] demonstrated that the transfer of the *PR1* gene into over-expressed *Arabidopsis* enhanced the resistance to rape downy mildew, and the induced expression of the gene was closely related to the accumulation of salicylic acid. Sarowar et al. [13] transferred the pepper *PR1* gene into tobacco, which enhanced the resistance to not only heavy metal stress but also to various pathogens such as *Phytophthora parasitica* and *Pseudomonas syringae* pv. tabaci. Plant *PR1* genes are polygenic; however, only some genes participate in disease resistance. A study reported that only the *VvPR1b1* gene in grape plants was involved in the resistance to *P. syringae* pv.tabaci [14]. Bonasera et al. [15] transferred *PR-1a*, *PR-1b*, and *PR-1c* into apple seedlings and found no disease resistance. In addition to their involvement in disease resistance, *PR* genes play an important role in normal plant growth, abiotic stress response, anti-aging, and hormone induction. The transcriptional levels of *PR1*, *PR2*, and *WRKY41* in *Arabidopsis* are significantly increased under drought stress [16], and those of *PR-5c* are increased in tobacco under salt stress [17]. In rice, the expression of the *OsPR1a* and *OsPR1b* genes can be induced by jasmonic acid (JA), salicylic acid (SA), hydrogen peroxide (H_2_O_2_), the protease inhibitor cantharidin (CN), and *Magnaporthe grisea*, and these genes are involved in the response to chemical and environmental stresses such as light, injury, and exposure to phosphatase inhibitors [18]. In this preliminary study, we identified a disease course-related gene, named *GmPR1*, in soybean using the Jinong 18 mutant (M18) transcriptomic library. The cDNA of *GmPR1L* with the complete coding region was cloned using M18 and introduced into soybean to detect the resistance to *C. sojina* and the activity of defense-related enzymes in transgenic soybean plants. Altogether, this study highlights the role of *GmPR1L* in resistance to fungal diseases and proposes an effective strategy for developing disease-resistant soybean cultivars.

Several studies have reported that PR proteins are involved in resistance to diverse biological stresses. Yamamoto et al. demonstrated that overexpression of *PR2* alone or in combination with *PR3* enhanced the resistance to multi-fungal infection [19]. Dai et al. reported that overexpression of the *PR4* gene in *Vitis vinifera* enhanced the resistance to powdery mildew infection [20]. Liu et al. proposed that another important member of the PR protein family is *PR5* or thaumatin-like protein, which is considered an important antimicrobial agent [21]. When overexpressed in tobacco or wheat plants, *PR5* exhibits increased resistance to a wide range of pathogens. Consistently, Chye et al. reported that overexpression of *PR12* and *PR13* enhanced the resistance to a wide range of pathogens. In particular, overexpression of *PR13* in tomato and potato plants enhanced the resistance to fungal pathogens [22]. These studies reflect substantial research progress of plant–pathogen interactions.

## 2. Results

### 2.1. Cloning and Bioinformatic Analysis of GmPR1L

**Figure 1:**
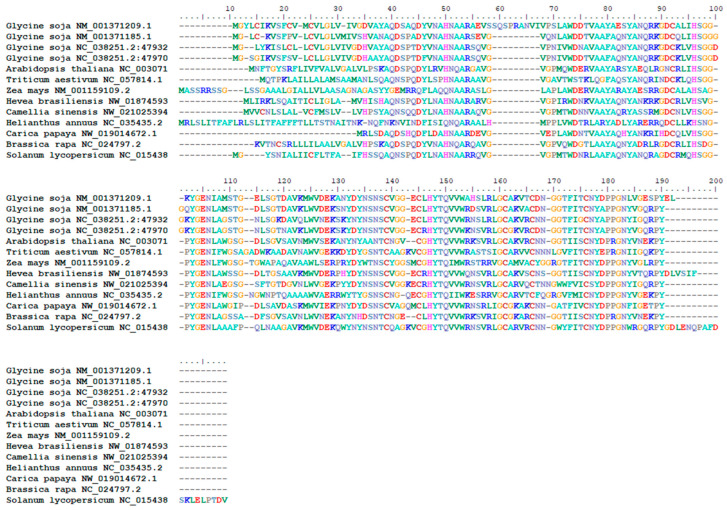
Gene similarity comparison between *GmPR1L* and multi-species *PR1* Family. *Glycine soja* NM_001371209.1: *GmPR1L* Target gene; *Glycine soja* NM_001371185.1: pathogenesis-related protein 1 (*PR1-6*); *Glycine soja* NC_038251.2:4793226-4794056: pathogenesis-related protein 1 (*PR1-7*); *Glycine soja* NC_038251.2:4797098-4797858: pathogenesis-related protein 1 (*PR1-8*); *Arabidopsis thaliana* NC_003071.7: pathogenesis-related protein 1 (*AtPR-1*); *Triticum aestivum* NC_057814.1: pathogenesis-related protein 1 (*TrPR-1*); *Zea mays* NM_001159109.2: pathogenesis-related protein (*ZmPR-1*); *Hevea brasiliensis* NW_018745933.1: *Hevea brasiliensis* *PR-1*; *Camellia sinensis* NW_021025394.1: pathogenesis-related protein (*Camellia sinensis PR-1*); *Helianthus annuus* NC_035435.2: pathogenesis-related protein *Helianthus annuus PR-1*; *Carica papaya* NW_019014672.1: pathogenesis-related protein (*Carica papaya PR-1*); Brassica rapa NC_024797.2: pathogenesis-related protein (*Brassica rapa PR-1*); *Solanum lycopersicum* NC_015438: pathogenesis-related protein (*Solanum lycopersicum PR-1*).

Using the soybean M18 leaf cDNA as a template, a specific target band was obtained via PCR amplification (Figure 1). The sequence encoded 174 aa residues and had a total length of 525 bp. The nucleotide sequence of the identified gene was compared with that of multiple *PR1* family genes of soybean, *Arabidopsis*, wheat, and maize (Figure 1). The identified sequence had the highest similarity with the sequence of *PR1-9* gene in soybean (registration number: NM_001371209.1). Therefore, the identified gene was named *GmPR1-like*, abbreviated as *GmPR1L*. In addition, the sequence of *GmPR1L* was >60% homologous to that of NM_001371185.1 (*PR1-6*), NC_038251.2:4793226-4794056 (*PR1-7*), and NC_038251.2:4797098-4797858 (*PR1-8*) in soybean.

**Figure 2:**
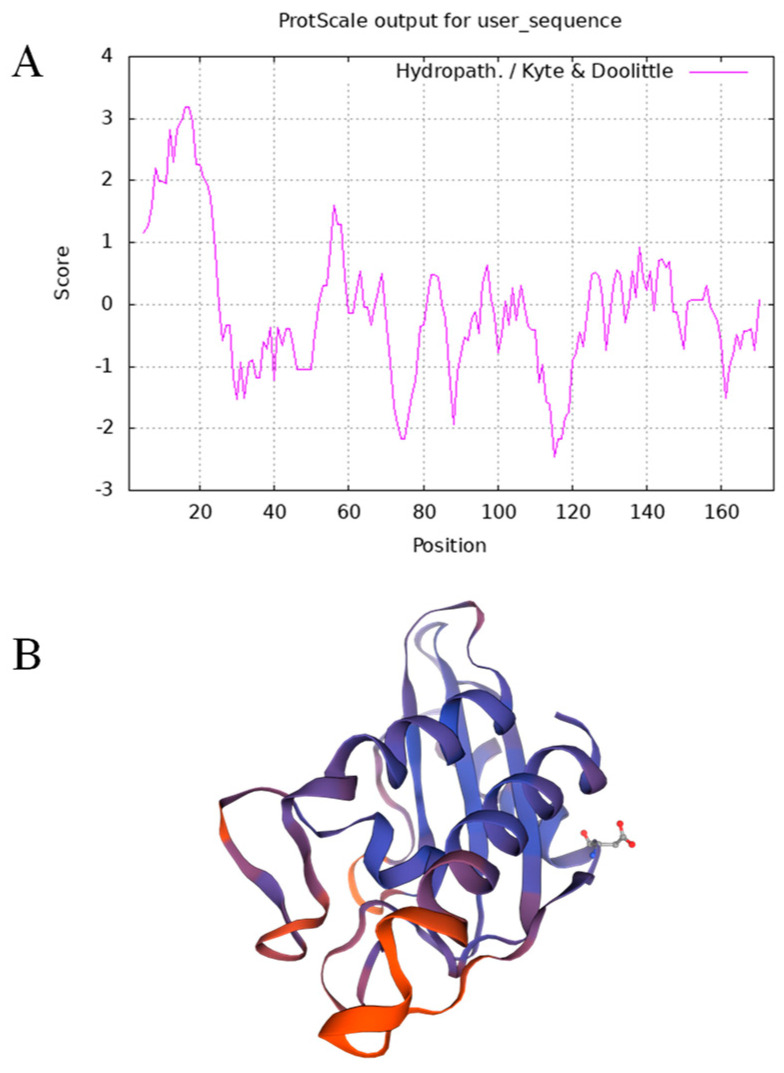
Bioinformatics analysis of *GmPR1L*. (**A**) Hydrophilicity and hydrophobicity of *GmPR1L* protein; (**B**) The tertiary structure of *GmPR1L* protein.

Isoleucine (Ile) at position 16 had the highest score (3.189) and high hydrophobicity, whereas arginine (Arg) at position 115 had the lowest score (−2.467) and the strongest hydrophilicity (Figure 2A). Overall, the number of hydrophilic amino acids was higher than that of hydrophobic amino acids in the sequence of *GmPR1L*, indicating that *GmPR1L* is hydrophilic. The three-dimensional structure of the *GmPR1L* protein is shown in Figure 2B.

### 2.2. Acquisition of the GmPR1L Expression Vector and Production of Transgenic Soybean Plants

The gene overexpression vector pCAMBIA3301-*GmPR1L*-over and the gene silencing vector pCAMBIA3301-*GmPR1L*-RNAi were successfully constructed through restriction enzyme digestion of BglⅡ and BstEⅡ. After PCR detection, the *GmPR1L*-overexpressing transgenic lines OEA1 and OEA2 and the *GmPR1L*-silenced lines IEA1 and IEA2 were obtained, which were added to T2 for subsequent functional identification.

### 2.3. Southern Blot Detection of Transgenic Soybean Plants

**Figure 3:**
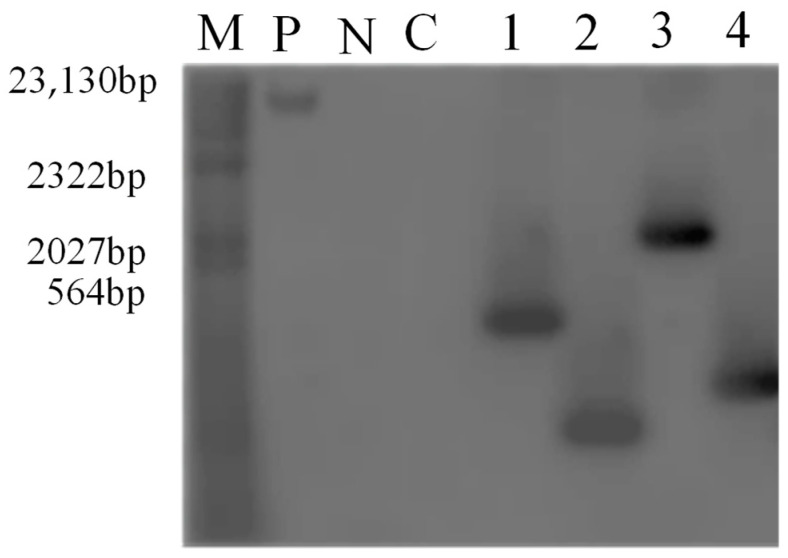
Southern blot detection of T2 transgenic plants. M: molecular weight standard. P: *GmPR1L* Overexpression Vector Plasmid N: water negative control C: untransformed plants 1–2: OEA1 and OEA2 overexpression positive transformed plants 3–4: IEA1 and IEA2 interference expression positive transformed plants.

According to the results of southern hybridization, the four transgenic plants showed hybrid fragments at different sites, with different sheet lengths, and in single copy forms (Figure 3), indicating that the exogenous screening marker Bar was successfully integrated into the plant genome.

### 2.4. Identification of the Phenotype of Soybean Plants Overexpressing the GmPR1L Gene during C. sojina Infection

**Figure 4:**
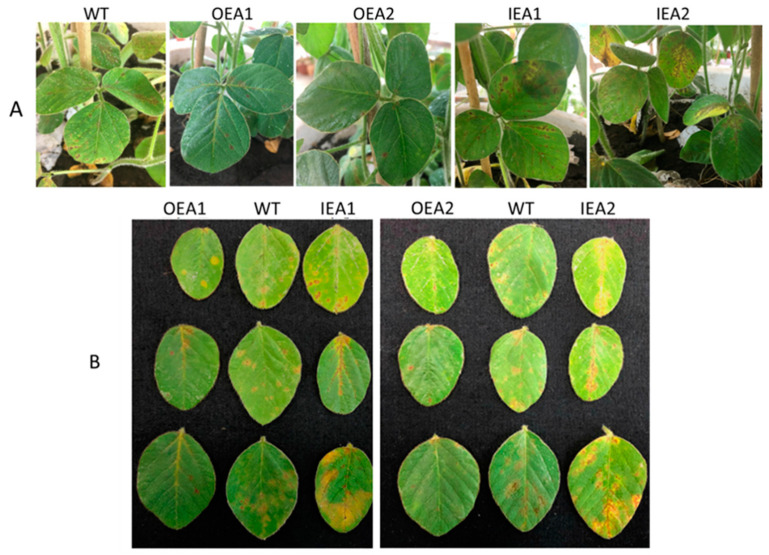
Identification of *Cercospora sojina* Hara resistance. (**A**) After 7 days of *C. sojina* pathogen infection, the untransformed receptor control WT, overexpression line OEA1-OEA2 and plant phenotype interference expression IEA1-IEA2 with the overall phenotype of plants. (**B**) After 7 days of *C. sojina* pathogen infection, the untransformed receptor control WT, overexpression line OEA1-OEA2, and leaf phenotype of interference expression IEA1–IEA2.

As shown in Figure 4A,B, evident rust spots gradually appeared on leaves after 7 days of *C. sojina* infection in the transgenic plants. Compared with control (WT) plants, OEA1 and OEA2 plants had significantly smaller leaf lesions. In addition, leaves were green, and the severity of disease was relatively mild in OEA1 and OEA2 plants. However, IEA1 and IEA2 plants had several rust spots and a larger lesion area, and their leaves were yellow, green, and slightly dry.

### 2.5. Resistance of Transgenic Soybean Plants to C. sojina Infection

According to the disease severity of transgenic soybean plants, the disease-related statistics of all transgenic plants are shown in Table 1. The results revealed that the disease index of the control line M18 was 50.22%, reaching a moderate level. The disease index of OEA1 and OEA2 plants was 37.78% and 35.56%, respectively, which improved from the moderate susceptibility level of the control plants to the moderate resistance level. The disease index of IEA1 and IEA2 plants was 62.86% and 62.54%, respectively, indicating the susceptibility of these plants to *C. sojina* infection. These results suggest that overexpression of *GmPR1L* can improve the resistance of soybean plants to *C. sojina* infection.

### 2.6. Determination of Disease Resistance and the Activity of Defense-Related Enzymes in Different Transgenic Soybean Lines

**Figure 5:**
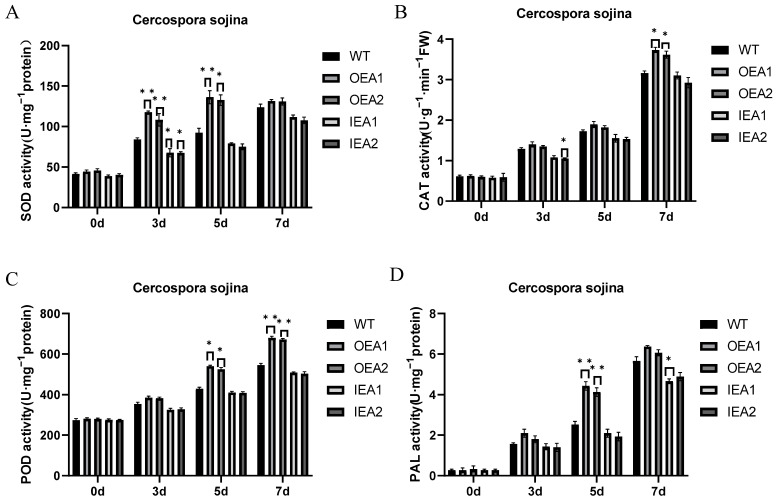
Defense enzyme activities of transgenic soybean lines infected by *Cercospora sojina* Hara. (**A**) Changes of SOD activity in OEA1-OEA2 overexpression line and the IEA1-IEA2 interference expression line after 0, 3, 5, and 7 days of *C. sojina* infection; (**B**) Changes of CAT activity in OEA1-OEA2 overexpression line and IEA1-IEA2 interference expression line after 0, 3, 5, and 7 days of *C. sojina* infection; (**C**) Changes of POD activity in OEA1-OEA2 overexpression line and IEA1-IEA2 interference expression line after 0, 3, 5, and 7 days of *C. sojina* infection; (**D**) Changes of PAL activity in OEA1-OEA2 overexpression line and IEA1-IEA2 interference expression line after 0, 3, 5, and 7 days of *C. sojina* infection. (* *p* < 0.05, ** *p* < 0.01).

When plants are infected by pathogens and encounter other biological stresses, the activity of defense-related enzymes is altered to improve resistance to the corresponding stresses. In this study, the activities of SOD, POD, CAT, and PAL were measured in transgenic soybean plants to examine the role of these enzymes in disease resistance. The results revealed that SOD activity was not significantly different among the five plant lines before *C. sojina* infection (Figure 5A). After 3 days of infection, SOD activity was significantly higher in OEA1 and OEA2 plants than in control plants. After 5 days of infection, SOD activity remained significantly higher in OEA1 and OEA2 plants than in control plants; however, it was significantly lower in IEA1 plants than in control plants. After 7 days of infection, SOD activity was extremely significantly higher in OEA1 plants and significantly higher in OEA2 plants than in control plants, whereas it was slightly lower in IEA1 and IEA2 plants than in control plants without significant differences. 

No significant difference was observed in POD activity among the five plant lines before *C. sojina* infection (Figure 5B). After 3 days of infection, POD activity was slightly higher in OEA1 and OEA2 plants than in control plants and slightly lower in IEA1 and IEA2 plants than in control plants; however, the differences were not significant. After 5 days of infection, POD activity was significantly higher in OEA1 and OEA2 plants than in control plants, whereas it was lower in IEA1 and IEA2 plants than in control plants. After 7 days of infection, POD activity was significantly higher in OEA1 and OEA2 plants than in control plants, whereas it was slightly lower in IEA1 and IEA2 plants than in control plants. 

No significant difference was observed in CAT activity among the five plant lines before *C. sojina* infection (Figure 5C). After 3 days of infection, CAT activity was slightly higher in OEA1 and OEA2 plants than in control plants, whereas it was significantly lower in IEA2 plants than in control plants. After 5 days of infection, CAT activity was slightly higher in OEA1 and OEA2 plants than in control plants, whereas it was significantly lower in IEA1 and IEA2 plants than in control plants, which had not reached a significant level. After 7 days of infection, CAT activity was significantly higher in OEA1 and OEA2 plants than in control plants, whereas it was slightly lower in IEA1 and IEA2 plants than in control plants.

No significant difference was observed in PAL activity among the five plant lines before *C. sojina* infection (Figure 5D). After 3 days of infection, PAL activity was slightly higher in OEA1 and OEA2 plants than in control plants, whereas it was slightly lower in IEA1 and IEA2 plants than in control plants. However, the differences were not significant. After 5 days of infection, PAL activity was significantly higher in OEA1 and OEA2 plants than in control plants. After 7 days of infection, PAL activity was slightly higher in OEA1 and OEA2 plants than in control plants, whereas it was significantly lower in IEA1 plants than in control plants. 

The abovementioned results suggest that overexpression of *GmPR1L* can improve the resistance of soybean plants to *C. sojina* infection.

### 2.7. Overexpression of GmPR1L Induced the Expression of Disease-Resistant Genes in Transgenic Soybean Plants

**Figure 6:**
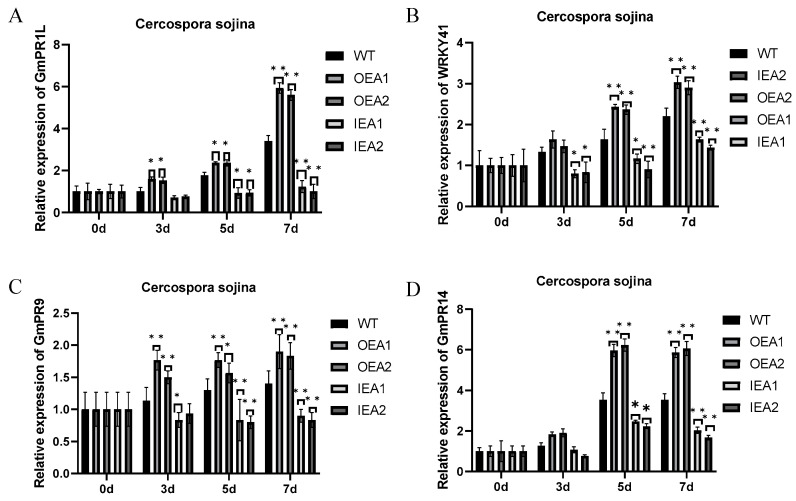
Determination of relative expression levels of *GmPR1L* and related endogenous genes in transgenic soybean lines infected by *Cercospora sojina* Hara. (**A**) Determination of relative expression of *GmPR1L* in overexpression lines OEA1-OEA2 and interference expression lines IEA1-IEA2 at 0, 3, 5, and 7 days of *C. sojina* infection; (**B**) Determination of relative expression of *WRKY* in OEA1-OEA2 and IEA1-IEA2 were measured at 0, 3, 5, and 7 days of *C. sojina* infection; (**C**) Determination of relative expression of *GmPR9* in the overexpression lines OEA1-OEA2 and the interference expression lines IEA1-IEA2 at 0, 3, 5, and 7 days of *C. sojina* infection.; (**D**) Determination of relative expression of *GmPR14* in overexpression lines OEA1-OEA2 and interference expression lines IEA1-IEA2 at 0, 3, 5, and 7 days of *C. sojina* infection (* *p* < 0.05, ** *p* < 0.01).

The relative expression of *GmPR1L* in the five soybean plant lines was measured before and after *C. sojina* infection (Figure 6A). The results revealed that the expression of *GmPR1L* was not different among the plant line before infection. After 3 days of infection, the relative expression of *GmPR1L* was significantly higher in OEA1 and OEA2 plants than in control plants, whereas it was slightly lower in IEA1 and IEA2 plants than in control plants, without a significant difference. After 5 days of infection, the relative expression of *GmPR1L* was significantly higher in OEA1 and OEA2 plants than in control plants, whereas it was significantly lower in IEA1 and IEA2 plants than in control plants. After 7 days of infection, the relative expression of *GmPR1L* was extremely significantly higher in OEA1 and OEA2 plants than in control plants, whereas it was extremely significantly lower in IEA1 and IEA2 plants than in control plants. These results indicated that the relative expression of *GmPR1L* gradually increased with the prolongation of the infection period in OEA1 and OEA2 plants. In particular, the expression of *GmPR1L* increased moderately during 0–5 days of infection but increased substantially after 7 days of incubation. Altogether, soybean plants with overexpression of *GmPR1L* had the strongest resistance to *C. sojina* infection.

Similarly, the relative expression of *WRKY41* was measured in the five soybean plant lines (Figure 6B). After 3 days of infection, the expression of *WRKY41* was significantly lower in IEA1 and IEA2 plants than in control plants. After 5 days of infection, the expression of WRKY41 was significantly higher in OEA1 and OEA2 plants than in control plants, whereas it was significantly lower in IEA1 plants and extremely significantly lower in IEA2 plants than in control plants. After 7 days of infection, the expression of *WRKY41* was significantly higher in OEA1 and OEA2 plants than in control plants, whereas it was extremely significantly lower in IEA1 and IEA2 plants than in control plants. These results indicated that overexpression of *GmPR1L* promoted the expression of *WRKY41* in transgenic soybean plants under biological stress. 

Owing to the overexpression of the target gene *GmPR1L* in soybean plants, the related resistance genes were activated after *C. sojina* infection. The expression of *GmPR9* was measured in the five soybean plant lines (Figure 6C). After 3 days of infection, the expression of *GmPR9* was significantly higher in OEA1 plants and significantly lower in IEA1 plants than in control plants. After 5 days of infection, the expression of *GmPR9* was significantly higher in OEA1 and OEA2 plants and extremely significantly lower in IEA1 plants than in control plants. After 7 days of infection, the expression of *GmPR9* was significantly higher in OEA1 and OEA2 plants and extremely significantly lower in IEA1 and IEA2 plants than in control plants. These results suggested that overexpression of *GmPR1L* induced the expression of *GmPR9* in soybean plants under biological stress. *GmPR9* was continuously and stably expressed from the initial stage of *C. sojina* infection to day 7, when the infection was most serious, thus improving the ability of transgenic plants to resist biological stress.

Furthermore, the relative expression of the disease duration-related gene *GmPR14* was measured in transgenic plants (Figure 6D). After 3 days of infection, the expression of *GmPR14* was not significantly different among the five plant lines. After 5 days of infection, the expression of *GmPR14* was extremely significantly higher in OEA1 and OEA2 plants and significantly lower in IEA1 and IEA2 plants than in control plants. After 7 days of infection, the expression of *GmPR14* was extremely significantly higher in OEA1 and OEA2 plants than in control plants. These results indicated that overexpression of *GmPR1L* induced the expression of *GmPR14* in transgenic soybean plants under biological stress and the expression of *GmPR1L* in transgenic lines remained high after 5 days of infection. Altogether, the results indicated that overexpression of *GmPR1L* in transgenic plants induced the upregulated expression of *WRKY41*, *GmPR9*, and *GmPR14* after *C. sojina* infection. *GmPR9* was expressed in the earliest stage of infection, and its expression was significantly higher in OEA1 and OEA2 plants than in control plants after 3 days of infection and peaked after 7 days of infection. *GmPR14* expression began to significantly increase after 5 days of infection and remained stable after 7 days of inoculation compared with that after 5 days of infection.

### 2.8. Statistical Analysis of Agronomic Traits of Transgenic Soybean Lines

The agronomic characteristics of transgenic soybean plants were investigated 120 days after the entire growth period from sowing to harvest (Table 2). The height of OEA1 plants was significantly higher than that of control plants. The height of IEA1 and IEA2 plants was slightly lower than that of control plants; however, the difference was not significant. Furthermore, no significant differences were observed in the number of branches and main stem nodes between the transgenic and control plants. The total pod number was significantly higher in OEA2 plants and slightly lower in IEA1 and IEA2 plants than in control plants. The four pod number was significantly higher in OEA1 plants than in control plants. The 100-grain weight of OEA1 plants was significantly higher than that of control plants, whereas the 100-grain weight of IEA1 and IEA2 plants was significantly lower than that of control plants. The growth period, leaf type, flower color, and seed umbilical color of all lines were consistent. These results demonstrated that overexpression of *GmPR1L* resulted in small improvements in plant height, total pod number per plant, and 100-grain weight in soybean plants.

## 3. Discussion

### 3.1. Relationship between Changes in the Activity of Defense-Related Enzymes and Disease Resistance

Peroxidase, cinnamic acid 4-hydroxylase, and 4-coumarin acetyl coenzyme A ligase are key enzymes involved in the phenylpropanoid metabolic pathway in plants. This pathway is closely associated with the synthesis of defense-related substances such as lignin and phytoalexin. Phenylalanine ammonia-lyase (PAL) is a rate-limiting enzyme in the metabolism of phenylpropanoid substances in plants. Infection caused by pathogenic bacteria and treatment with pathogenic toxins can induce an increase in the activity of PAL, which is positively correlated with disease resistance.

Superoxide dismutase (SOD), an important reactive oxygen species scavenger in plants [23], can greatly reduce the toxicity induced by excessive accumulation of reactive oxygen species so as to protect the cell membrane from damage [24,25,26,27]. Abiotic stresses such as drought, saline, cold injury, and endogenous and exogenous phytohormones [28] and biotic stresses such as pathogenic bacteria can lead to changes in SOD activity [29,30,31]. Du et al. [32] demonstrated that overexpression of hrpzm() resulted in different degrees of increase in SOD activity in transgenic soybean plants, and multiple peaks appeared, which enhanced the resistance of transgenic soybean to Phytophthora root rot [33]. This study demonstrated that SOD activity was significantly or extremely significantly higher in OEA1 and OEA2 plants and significantly lower in IEA1 and IEA2 plants than in control plants after *C. sojina* infection. With the increase in infection time, SOD activity gradually increased and peaked after 7 days of infection. These results indicate that overexpression of *GmPR1L* can improve the resistance of soybean plants to *C. sojina* infection by increasing SOD activity. 

Catalase (CAT) is one of the most important enzymes for scavenging reactive oxygen species in plants, mainly in the glyoxylate cycle and peroxidation cycle pathways [34,35,36,37]. Gao et al. [38] demonstrated that CAT activity in the leaves of grape plants was significantly altered after the plants were subjected to *Apolygus lucorum* infection. Consistently, this study showed that CAT activity peaked during 5–7 days of infection with *C. sojina* in *GmPR1L*-overexpressing soybean plants. CAT activity was higher in OEA1 and OEA2 plants but lower in IEA1 and IEA2 plants than in control plants. These results indicate that overexpression of *GmPR1L* can improve the resistance of soybean to *C. sojina* infection by increasing CAT activity. 

Peroxidase (POD) is another important defense-related enzyme in plant cells. It not only participates in scavenging reactive oxygen species [39,40,41,42] but also plays a role in the synthesis of lignin. In addition, POD activity is closely related to the synthesis of phytoprotectin and the oxidation of phenolic substances [43]. The high lignification of the cell wall has a certain limiting effect on the invasion and spread of pathogenic bacteria. This study demonstrated that POD activity was significantly higher in OEA1 and OEA2 plants but lower in IEA1 and IEA2 plants than in control plants on days 5 and 7 of infection. These results indicate that overexpression of *GmPR1L* increases the activity of POD, thus improving the resistance of soybean plants to *C. sojina* infection. 

When plants are infected by pathogens, numerous phenolic substances synthesized through shikimic acid or acetic acid metabolism are accumulated in the plant body. PAL is a key enzyme involved in the metabolism of shikimic acid metabolism [44,45] and also in the anabolism of phenolic substances. Previous studies have reported that PAL activity is significantly increased in most resistance responses owing to incompatible interactions. Gayoso et al. [46] demonstrated that the activities of PAL and POD and the content of lignin in verticillium wilt-resistant tomatoes were increased after the plants were infected with the causative agent of verticillium wilt. This study revealed that PAL activity began to increase in transgenic overexpression lines after 3 days of *C. sojina* infection. PAL activity was significantly higher in OEA1 and OEA2 plants than in control plants after 5 days of infection, whereas it was significantly lower in IEA1 and IEA2 plants than in control plants after 7 days of infection. PAL activity substantially increased in the early stage of infection. These results indicate that overexpression of *GmPR1L* can increase PAL activity, thereby reducing the production of phenolic substances and improving the resistance of soybean plants to *C. sojina* infection. 

### 3.2. Analysis of Expression Patterns of Disease Resistance-Related Endogenous Genes

Numerous studies have validated that transcription factors are important factors affecting biotic and abiotic stresses in plants [47,48,49]. The main transcription factors involved in disease resistance in plants include *WRKY*, *MYB*, *ZFP*, and *ZIP*. Among these, *WRKY* has the most positive response [50]. *P. syringae* infection induces the expression of *AtWRKY41*, which regulates *AtPR5* and induces the expression of *AtPDF1.2*, indicating that *AtWRKY41* participates in SA and JA signal transduction to regulate the response of plants to pathogens. This study demonstrated that *GmPR1L* expression gradually increased with an increase in infection time in OEA1 and OEA2 plants and peaked on day 7 of infection. The expression pattern of *WRKY41* was consistent with that of *GmPR1L* in *GmPR1L*-overexpressing plants. After 7 days of inoculation, the expression of *WRKY41* was significantly higher in OEA1 and OEA2 plants and significantly lower in IEA1 and IEA2 plants than in control plants, indicating that overexpression of *GmPR1L* promoted the expression of *WRKY41*. Among PR family genes, *PR9* and *PR14* are important genes involved in the resistance of soybean to fungal diseases, and their expression is positively regulated by the ethylene (ETH) synthesis signal during stress response. In addition, *PR9* expression is related to the auxin (AUX) synthesis signal. Overexpressed *PR9* and *PR14* are involved in the formation of antimicrobial substances, such as lignin, and other major components of the plant cell wall or cuticle. Therefore, the upregulated expression of *PR9* and *PR14* enhances the ability of plants to defend against disease. This study consistently demonstrated that the expression of *GmPR9* and *GmPR14* was significantly higher in *GmPR1L*-overexpressing plants than in control plants. In *GmPR1L*-overexpressing plants, the expression of *GmPR9* began to increase significantly after 3 days of infection, whereas that of *GmPR14* began to increase significantly after 5 days of inoculation. These results indicate that overexpression of *GmPR1L* can induce the upregulation of *GmPR9* and *GmPR14*, thereby improving the resistance of soybean to *C. sojina* infection.

## 4. Materials and Methods

### 4.1. Identification and Sequence Homology Analysis of the Target Gene GmPR1L

An M18 pathogenesis-related gene was detected via RNA-seq. GmPR1L S/*GmPR1L*AS was cloned as a specific primer for this gene. The primer sequences are shown in Table S1. In addition, the pMD-18T-*GmPR1L* cloning vector was constructed. Homologous sequences were searched according to the nucleotide sequences of target genes in the NCBI database, and sequence similarity was compared using DNAMAN8.0.

### 4.2. Construction of GmPR1 L Overexpression Vector and Genetic Transformation

The CE Design software was used to design the overexpression homologous-arm primer *GmPR1L*-overS/*GmPR1L*-overAS to construct the overexpression vector pCAMBIA3301-*GmPR1L*-over. The *GmPR1L*-zS, *GmPR1L*-*zASGmPR1L*-fS, *GmPR1L*-fAS, *GmPR1L*-nS, and *GmPR1L*-nAS primers were designed to construct the RNAi interference vector pCAMBIA3301-*GmPR1L*-RNAi. The specific sequences of the abovementioned primers are shown in Table S1. The soybean cotyledon node was transformed into M18 through *Agrobacterium*-mediated transformation. The transformed plants were detected via PCR until T2-generation positive plants were obtained. The marker gene Bar was used as a probe and the *GmPR1L* overexpression vector was used as a positive control for southern blotting. Subsequently, the expression of disease resistance-related genes was evaluated. 

### 4.3. Bioinformatic Analysis of GmPR1L

Based on the full-length sequence of the *GmPR1L* gene, the online website ORF Finder was used to obtain its open reading frame and amino acid sequence. The online software SWISS-MODEL was used to construct the tertiary structure of the protein based on the amino acid sequence, and ProtScale was used to analyze the hydrophilicity and hydrophobicity of the protein.

### 4.4. Infection of Transgenic Soybean Plants with C. sojina

Experimental materials were strictly selected, and impurities were removed. Uniform and healthy T2-generation *GmPR1L*-overexpressing (OEA1 and OEA2) plants, *GmPR1L*-silenced (IEA1 and IEA2) plants, and control (M18) plants were selected for identification of plants successfully infected with *C. sojina* at the first flowering stage. Each line was sampled thrice. For bacterial species propagation, PDA was used to propagate *C. sojina* at 25 °C for 20 days. For infection, the leaf surface was sprayed with water containing *C. sojina*, and plants were selected for verifying successful infection before the flowering period. A high-power microscope was used for analysis. After the number of spores was approximately 20 under a 100-fold field of view, the plants were sprayed with the infective agent. The humidity was >90%, and the temperature was approximately 25 °C. 

### 4.5. Evaluation of the Resistance of Transgenic Soybean to C. sojina Infection

According to NY/T495-2002 “Technical Specifications for Identification of *C. sojina*”, the disease-related parameters of plants were evaluated at different time points during seven days of infection. In addition, the lesion area on plant leaves was evaluated and recorded to determine the disease severity. The disease index (DI) was calculated using the following Formula (1) to determine the resistance of plants to gray spot disease.

(1) $$\begin{array}{l} \;\;\;\mathrm{Disease}\;\mathrm{index}\;(\mathrm{DI})=\\ \frac{\Sigma \;\left(\mathrm{Representative}\;\mathrm{value}\;\mathrm{of}\;\mathrm{incidence}\;\mathrm{level}\;\times \;\mathrm{number}\;\mathrm{of}\;\mathrm{diseased}\;\mathrm{plants}\;\mathrm{of}\;\mathrm{this}\;\mathrm{level}\right)\times 100\%}{\left(\mathrm{Total}\;\mathrm{number}\;\mathrm{of}\;\mathrm{investigated}\;\mathrm{plants}\;\times \;\mathrm{representative}\;\mathrm{value}\;\mathrm{of}\;\mathrm{the}\;\mathrm{highest}\;\mathrm{incidence}\;\mathrm{level}\right)} \end{array}$$

In the abovementioned equation, Σ represents the sum of the product values of all levels.

### 4.6. Determination of the Activity of Defense-Related Enzymes in Transgenic Soybean Plants Infected with C. sojina

The receptor material with three fully expanded compound leaves at the seedling stage and different transgenic soybean lines with either overexpression or suppression of *GmPR1L* gene were used to examine disease resistance. The leaves of these plants were used to determine the activity of various defense-related enzymes. SOD activity was determined via nitrogen blue tetrazole (NBT) photoreduction. POD activity was determined using the guaiacol method. The activities of CAT and PAL were measured via spectrophotometry. Each experiment was repeated thrice. Statistical analysis was performed using the SPSS Statistics (version 11.0) software. 

### 4.7. Analysis of the Expression Pattern of GmPR1L Gene and Related Endogenous Genes in Plants Infected with C. sojina

After 3 days, 5 days, and 7 days of *C. sojina* infection, total RNA was extracted from the leaves of the infected plants for fluorescence quantitative detection, and the relative expression of the target gene *GmPR1L* in transgenic plants was further analyzed. The total RNA of the recipient material M18 was extracted using an RNA extraction kit. cDNA was synthesized using a reverse transcription kit, and the relative mRNA expression of *GmPR1L*, *WRKY*, *PR9*, and *PR14* was determined via qRT-PCR. The primers used for PCR included QT-*GmPR1L*S/QT-*GmPR1L*AS, QT-*WRKY*S/QT-*WRKY*AS, QT-*PR9*S/QT-*PR9*AS, and QT-*PR14*S/QT-*PR14*AS. Actin2S and Actin2AS were used as internal reference primers. The sequences of all abovementioned primers are shown in Table S1.

### 4.8. Agronomic Trait Analysis of Transgenic Soybean Lines Resistant to C. sojina

Completely positive soybean plants were collected from the field, with five plants collected from each line. The plant height, branch number, main stem node number, total pod number per plant, four pod number, 100-grain weight, growth period, leaf type, flower color, and umbilical color were examined. The SPSS Statistics (version 11.0) software was used for statistical analysis. One-factor analysis of variance was used for estimating differences between groups. A *p*-value of <0.05 indicated significant differences, and a *p*-value of <0.01 indicated extremely significant differences. 

## 5. Conclusions

Overexpression of *GmPR1L* can improve the ability of soybean plants to scavenge reactive oxygen species and enhance cell wall lignification during *C. sojina* infection. In addition, it can accelerate the response of plants to biological stress, thereby transmitting signals to pathways associated with hormone metabolism and inducing the expression of multiple resistance-related genes to improve the resistance of soybean plants to *C. sojina* infection.

## Figures and Tables

**Table 1:** Statistics of *Cercospora sojina* Hara.

Lines	Total Number of Investigations	Disease Level						Disease Index	Resistance Evaluation
		0	1	3	5	7	9		
WT	45	2	13	25	5	0	0	50.22	MS
OEA1	45	8	16	18	3	0	0	37.78	MR
OEA2	45	7	19	17	2	0	0	35.56	MR
IEA1	45	3	4	5	26	7	0	62.86	S
IEA2	45	2	3	9	25	6	0	62.54	S

**Table 2:** Agronomic traits analysis of the transgenic plants.

Genotype	WT	OEA1	OEA2	IEA1	IEA2
Plant height (cm)	85.4 ± 6.88	100 ± 7.04 *	99.4 ± 4.18 *	83.2 ± 8.42	81.8 ± 6.55
Branching number	3.6 ± 1.14	4.8 ± 1.17	4 ± 0.71	2.2 ± 1.09	3.4 ± 1.52
Node number	17.4 ± 1.95	19.4 ± 2.3	19.2 ± 1.30	15.6 ± 1.52	15 ± 3.08
Total pods per plant	80.4 ± 28.31	165.6 ± 90.83	136 ± 33.29 *	73.8 ± 23.01	79.4 ± 38.11
Number of four pods	2.2 ± 2.17	4.8 ± 4.60 **	5.4 ± 3.91	4.4 ± 2.61	1.6 ± 1.14
100 seed weight (g)	17.34 ± 0.39	19.44 ± 1.31 *	18.71 ± 2.74	14.2 ± 1.51 **	14.32 ± 1.98 *
Maturity period (days)	124	124	124	124	124
Leaf shape	Round	Round	Round	Round	Round
Flower color	Purple	Purple	Purple	Purple	Purple
Hilum color	Black	Black	Black	Black	Black

Within a row and treatment (WT or transformed strain), values followed by asterisks are significantly different from WT (* *p* < 0.05, ** *p* < 0.01).

## Data Availability

Not applicable.

## Supplementary Materials

The following supporting information can be downloaded at: https://www.mdpi.com/article/10.3390/genes14040920/s1, Table S1. Primer sequence.

## References

[1] Kattupalli D., Srinivasan A., Soniya E. (2021). A Genome-Wide Analysis of Pathogenesis-Related Protein-1 (*PR-1*) Genes from *Piper nigrum* Reveals Its Critical Role during *Phytophthora capsici* Infection. Genes.

[2] Whitham S.A., Qi M., Innes R.W., Ma W., Lopes-Caitar V., Hewezi T. (2016). Molecular Soybean-Pathogen Interactions. Annu. Rev. Phytopathol..

[3] Breen S., Williams S.J., Outram M., Kobe B., Solomon P.S. (2017). Emerging Insights into the Functions of Pathogenesis-Related Protein 1. Trends Plant Sci..

[4] Baek D., Kim M.C., Kumar D., Park B., Cheong M.S., Choi W., Park H.C., Chun H.J., Park H.J., Lee S.Y. (2019). AtPR5K2, a PR5-Like Receptor Kinase, Modulates Plant Responses to Drought Stress by Phosphorylating Protein Phosphatase 2Cs. Front. Plant Sci..

[5] Arora R., Kumar A., Singh I.K., Singh A. (2020). Pathogenesis related proteins: A defensin for plants but an allergen for humans. Int. J. Biol. Macromol..

[6] van Verk M.C., Pappaioannou D., Neeleman L., Bol J.F., Linthorst H.J. (2008). A Novel WRKY transcription factor is required for induction of *PR-1a* gene expression by salicylic acid and bacterial elicitors. Plant Physiol..

[7] Kothari K.S., Dansana P.K., Giri J., Tyagi A.K. (2016). Rice Stress Associated Protein 1 (OsSAP1) Interacts with Aminotransferase (OsAMTR1) and Pathogenesis-Related 1a Protein (OsSCP) and Regulates Abiotic Stress Responses. Front. Plant Sci..

[8] Kong X., Zhang D., Pan J., Zhou Y., Li D. (2013). Hydrogen peroxide is involved in nitric oxide-induced cell death in maize leaves. Plant Biol..

[9] Rahman F.U., Khan I.A., Aslam A., Liu R., Sun L., Wu Y., Aslam M.M., Khan A.U., Li P., Jiang J. (2022). Transcriptome analysis reveals pathogenesis-related gene 1 pathway against salicylic acid treatment in grapevine (*Vitis vinifera* L.). Front. Genet..

[10] Mitsuhara I., Iwai T., Seo S., Yanagawa Y., Kawahigasi H., Hirose S., Ohkawa Y., Ohashi Y. (2008). Characteristic expression of twelve rice *PR1* family genes in response to pathogen infection, wounding, and defense-related signal compounds (121/180). Mol. Genet. Genom..

[11] Li Y., Qiu L., Liu X., Zhang Q., Zhuansun X., Fahima T., Krugman T., Sun Q., Xie C. (2020). Glycerol-Induced Powdery Mildew Resistance in Wheat by Regulating Plant Fatty Acid Metabolism, Plant Hormones Cross-Talk, and Pathogenesis-Related Genes. Int. J. Mol. Sci..

[12] Bonasera J.M., Kim J.F., Beer S.V. (2006). PR genes of apple: Identification and expression in response to elicitors and inoculation with Erwinia amylovora. BMC Plant Biol..

[13] Seo J.S., Diloknawarit P., Park B.S., Chua N.-H. (2019). ELF18-induced long noncoding RNA 1 evicts fibrillarin from mediator subunit to enhance pathogenesis-related gene 1 (PR1) expression. New Phytol..

[14] Ren X.-B., Dang Y.-R., Liu S.-S., Huang K.-X., Qin Q.-L., Chen X.-L., Zhang Y.-Z., Wang Y.-J., Li P.-Y. (2022). Identification and Characterization of Three Chitinases with Potential in Direct Conversion of Crystalline Chitin into *N*,*N*′-diacetylchitobiose. Mar. Drugs.

[15] Agrawal G.K., Rakwal R., Jwa N.S., Agrawal V.P. (2001). Signaling molecules and blast pathogen attack activates rice OsPR1a and OsPR1b genes: A model illustrating components participating during defence/stress response. Plant Physiol. Biochem..

[16] Lu S., Friesen T.L., Faris J.D. (2011). Molecular characterization and genomic mapping of the pathogenesis-related protein 1 (PR-1) gene family in hexaploid wheat (*Triticum aestivum* L.). Mol. Genet. Genom..

[17] Liu Q., Xue Q. (2006). Computational identification of novelPR-1-type genes in *Oryza sativa*. J. Genet..

[18] Gamir J., Darwiche R., Hof P.V., Choudhary V., Stumpe M., Schneiter R., Mauch F. (2017). The sterol-binding activity of Pathogenesis-Related Protein 1 reveals the mode of action of an antimicrobial protein. Plant J. Cell Mol. Biol..

[19] Yamamoto T., Iketani H., Ieki H., Nishizawa Y., Notsuka K., Hibi T., Hayashi T., Matsuta N. (2000). Transgenic grapevine plants expressing a rice chitinase with enhanced resistance to fungal pathogens. Plant Cell Rep..

[20] Dai L., Wang D., Xie X., Zhang C., Wang X., Xu Y., Wang Y., Zhang J. (2016). The Novel Gene VpPR4-1 from Vitis pseudoreticulata Increases Powdery Mildew Resistance in Transgenic *Vitis vinifera* L.. Front. Plant Sci..

[21] Zhang J., Wang F., Liang F., Zhang Y., Ma L., Wang H., Liu D. (2018). Functional analysis of a pathogenesis-related thaumatin-like protein gene *TaLr35PR5* from wheat induced by leaf rust fungus. BMC Plant Biol..

[22] Chye M.-L., Zhao K.-J., He Z.-M., Ramalingam S., Fung K.-L. (2005). An agglutinating chitinase with two chitin-binding domains confers fungal protection in transgenic potato. Planta.

[23] Sinha M., Singh R.P., Kushwaha G.S., Iqbal N., Singh A., Kaushik S., Kaur P., Sharma S., Singh T.P. (2014). Current Overview of Allergens of Plant Pathogenesis Related Protein Families. Sci. World J..

[24] Anuradha C., Chandrasekar A., Backiyarani S., Thangavelu R., Giribabu P., Uma S. (2022). Genome-wide analysis of pathogenesis-related protein 1 (*PR-1*) gene family from Musa spp. and its role in defense response during stresses. Gene.

[25] Sels J., Mathys J., De Coninck B.M., Cammue B.P., De Bolle M.F. (2008). Plant pathogenesis-related (PR) proteins: A focus on PR peptides. Plant Physiol. Biochem..

[26] Christensen A.B., Cho B.H., Naesby M., Gregersen P.L., Brandt J., Madriz-Ordeñana K., Collinge D.B., Thordal-Christensen H. (2002). The molecular characterization of two barley proteins establishes the novel PR-17 family of pathogenesis-related proteins. Mol. Plant Pathol..

[27] Bai S., Dong C., Li B., Dai H. (2013). A PR-4 gene identified from *Malus domestica* is involved in the defense responses against *Botryosphaeria dothidea*. Plant Physiol. Biochem..

[28] Ghorbel M., Zribi I., Missaoui K., Drira-Fakhfekh M., Azzouzi B., Brini F. (2021). Differential regulation of the durum wheat Pathogenesis-related protein (PR1) by Calmodulin TdCaM1.3 protein. Mol. Biol. Rep..

[29] Du Q., Yang X., Zhang J., Zhong X., Kim K.S., Yang J., Xing G., Li X., Jiang Z., Li Q. (2018). Over-expression of the Pseudomonas syringae harpin-encoding gene hrpZm confers enhanced tolerance to Phytophthora root and stem rot in transgenic soybean. Transgenic Res..

[30] AlHudaib K.A., Alanazi N.A., Ghorbel M., El-Ganainy S.M., Brini F. (2022). Isolation and Characterization of a Novel Pathogenesis-Related Protein-1 Gene (*AvPR-1*) with Induced Expression in Oat (*Avena sativa* L.) during Abiotic and Hormonal Stresses. Plants.

[31] Liu X., Huang B., Lin J., Fei J., Chen Z., Pang Y., Sun X., Tang K. (2006). A novel pathogenesis-related protein (SsPR10) from Solanum surattense with ribonucleolytic and antimicrobial activity is stress- and pathogen-inducible. J. Plant Physiol..

[32] Xie Y.-R., Chen Z.-Y., Brown R.L., Bhatnagar D. (2010). Expression and functional characterization of two pathogenesis-related protein 10 genes from Zea mays. J. Plant Physiol..

[33] Prasath D., El-Sharkawy I., Sherif S., Tiwary K.S., Jayasankar S. (2011). Cloning and characterization of PR5 gene from Curcuma amada and Zingiber officinale in response to Ralstonia solanacearum infection. Plant Cell Rep..

[34] Pečenková T., Pleskot R., Žárský V. (2017). Subcellular Localization of Arabidopsis Pathogenesis-Related 1 (PR1) Protein. Int. J. Mol. Sci..

[35] Gao S., Zhou G., Sun T., Liu J., Kong W., Wu H. (2022). Apolygus lucorum-induced resistance in Vitis vinifera L. elicits changes at the phenotypic, physiological, and biochemical levels. Sci. Hortic..

[36] Akbudak M.A., Yildiz S., Filiz E. (2020). Pathogenesis related protein-1 (PR-1) genes in tomato (*Solanum lycopersicum* L.): Bioinformatics analyses and expression profiles in response to drought stress. Genomics.

[37] Goyal R.K., Fatima T., Topuz M., Bernadec A., Sicher R., Handa A.K., Mattoo A.K. (2016). Pathogenesis-Related Protein 1b1 (PR1b1) Is a Major Tomato Fruit Protein Responsive to Chilling Temperature and Upregulated in High Polyamine Transgenic Genotypes. Front. Plant Sci..

[38] Liu T., Chen T., Kan J., Yao Y., Guo D., Yang Y., Ling X., Wang J., Zhang B. (2022). The GhMYB36 transcription factor confers resistance to biotic and abiotic stress by enhancing *PR1* gene expression in plants. Plant Biotechnol. J..

[39] Sung Y., Outram M.A., Breen S., Wang C., Dagvadorj B., Winterberg B., Kobe B., Williams S.J., Solomon P.S. (2021). PR1-mediated defence via C-terminal peptide release is targeted by a fungal pathogen effector. New Phytol..

[40] Zhang X., Ménard R., Li Y., Coruzzi G.M., Heitz T., Shen W.-H., Berr A. (2020). Arabidopsis SDG8 Potentiates the Sustainable Transcriptional Induction of the Pathogenesis-Related Genes *PR1* and *PR2* During Plant Defense Response. Front. Plant Sci..

[41] Hussain R.M.F., Sheikh A.H., Haider I., Quareshy M., Linthorst H.J.M. (2018). Arabidopsis WRKY50 and TGA Transcription Factors Synergistically Activate Expression of PR1. Front. Plant Sci..

[42] Bozbuga R. (2020). Expressions of Pathogenesis related 1 (PR1) Gene in Solanum lycopersicum and Influence of Salicylic Acid Exposures on Host-Meloidogyne incognita Interactions. Dokl. Biochem. Biophys..

[43] Gayoso C., Pomar F., Novo-Uzal E., Merino F., de Ilárduya Ó.M. (2010). The Ve-mediated resistance response of the tomato to Verticillium dahliae involves H_2_O_2_, peroxidase and lignins and drives PALgene expression. BMC Plant Biol..

[44] Guo J., Bai Y., Wei Y., Dong Y., Zeng H., Reiter R.J., Shi H. (2022). Fine-tuning of pathogenesis-related protein 1 (PR1) activity by the melatonin biosynthetic enzyme ASMT2 in defense response to cassava bacterial blight. J. Pineal Res..

[45] Oide S., Bejai S., Staal J., Guan N., Kaliff M., Dixelius C. (2013). A novel role of PR 2 in abscisic acid (ABA) mediated, pathogen-induced callose deposition in *Arabidopsis thaliana*. New Phytol..

[46] Park C.-J., Kim K.-J., Shin R., Park J.M., Shin Y.-C., Paek K.-H. (2004). Pathogenesis-related protein 10 isolated from hot pepper functions as a ribonuclease in an antiviral pathway. Plant J..

[47] Li R., Li Y., Zhang Y., Sheng J., Zhu H., Shen L. (2021). Transcriptome analysis reveals that SlNPR1 mediates tomato fruit resistance against *Botrytis cinerea* by modulating phenylpropanoid metabolism and balancing ROS homeostasis. Postharvest Biol. Technol..

[48] Luo X., Tian T., Feng L., Yang X., Li L., Tan X., Wu W., Li Z., Treves H., Serneels F. (2023). Pathogenesis-related protein 1 suppresses oomycete pathogen by targeting against AMPK kinase complex. J. Adv. Res..

[49] Ijaz S., Haq I.U., Khan I.A., Ali H.M., Kaur S., Razzaq H.A. (2022). Identification of resistance gene analogs of the NBS-LRR family through transcriptome probing and in silico prediction of the expressome of Dalbergia sissoo under dieback disease stress. Front. Genet..

[50] Lodhi N., Singh M., Srivastava R., Sawant S.V., Tuli R. (2023). Epigenetic malleability at core promoter initiates tobacco PR-1a expression post salicylic acid treatment. Mol. Biol. Rep..

