# Supplementary material for: Identification of the Function of the Pathogenesis-Related Protein GmPR1L in the Resistance of Soybean to Cercospora sojina Hara
Source: Genes (Basel). 2023 Apr 15;14(4):920. doi: 10.3390/genes14040920 (PMC10137329; doi:10.3390/genes14040920)
Supplement: Supplementary file 1 [file genes-14-00920-s001.zip › genes-2292008-supplementary.pdf]

Supplementary Table

Table S1. Primer sequence

Gene name	Primer sequence (5'-3')
*Gm*PR1L*L*S	ATAATGGGATACTTGTGC
*GmPR1LL*AS	CTACAGTTCGTAGGGACTT
Bars	TCAAATCTCGGTGACGGGC
Baras	CGGTCTGCACCATCGTCAA
35S	TAG AGG ACC TAA CAG AAC
35AS	CCG TGT TCT CTC CAA ATG
NosS	GAA TCC TGT TGC CGG TCT TG
NosAS	TTA TCC TAG TTT GCG CGC TA
QT- *GmPR1LL*S	TTGGGATGATACGGTTGCTG
QT- *GmPR1LL*AS	ASTGTAGTCATAGTTGGCTTTCTC
35s*GmPR1LL*nosS	AGGACCTAACAGAACTCGCCGT
35sGmPR1LLnosSAS	GACTCTAATCATAAAAACCCAT
GmPR1L-zS	ACTCTTGACCATGGTAGATCTATAATGGGATACTTGTGC
GmPR1L-zAS	CACAGATGCGTAAGGAGAAACTACAGTTCGTAGGGACTT
GmPR1L-fS	TCACCGTCTACAGTTCGTAGGGACTT
GmPR1L-fAS	GGGGAAATTCGAGCTGGTCACCATAATGGGATACTTGTGC
GmPR1L-nS	AACTGTAGTTTCTCCTTACGCATCTGTG
GmPR1L-nAS	TTGACATCACGGTGAAAACCTCTGACA
